# Targeting of *Drosophila* Rhodopsin Requires Helix 8 but Not the Distal C-Terminus

**DOI:** 10.1371/journal.pone.0006101

**Published:** 2009-07-02

**Authors:** Ines Kock, Natalia A. Bulgakova, Elisabeth Knust, Irmgard Sinning, Valérie Panneels

**Affiliations:** 1 Heidelberg University Biochemistry Center (BZH), INF328, Heidelberg, Germany; 2 Max-Planck Institute for Molecular Cell Biology and Genetics, Dresden, Germany; University of Oldenburg, Germany

## Abstract

**Background:**

The fundamental role of the light receptor rhodopsin in visual function and photoreceptor cell development has been widely studied. Proper trafficking of rhodopsin to the photoreceptor membrane is of great importance. In human, mutations in rhodopsin involving its intracellular mislocalization, are the most frequent cause of autosomal dominant Retinitis Pigmentosa, a degenerative retinal pathology characterized by progressive blindness. *Drosophila* is widely used as an animal model in visual and retinal degeneration research. So far, little is known about the requirements for proper rhodopsin targeting in *Drosophila*.

**Methodology/Principal Findings:**

Different truncated fly-rhodopsin Rh1 variants were expressed in the eyes of *Drosophila* and their localization was analyzed *in vivo* or by immunofluorescence. A mutant lacking the last 23 amino acids was found to properly localize in the rhabdomeres, the light-sensing organelle of the photoreceptor cells. This constitutes a major difference to trafficking in vertebrates, which involves a conserved QVxPA motif at the very C-terminus. Further truncations of Rh1 indicated that proper localization requires the last amino acid residues of a region called helix 8 following directly the last transmembrane domain. Interestingly, the very C-terminus of invertebrate visual rhodopsins is extremely variable but helix 8 shows conserved amino acid residues that are not conserved in vertebrate homologs.

**Conclusions/Significance:**

Despite impressive similarities in the folding and photoactivation of vertebrate and invertebrate visual rhodopsins, a striking difference exists between mammalian and fly rhodopsins in their requirements for proper targeting. Most importantly, the distal part of helix 8 plays a central role in invertebrates. Since the last amino acid residues of helix 8 are dispensable for rhodopsin folding and function, we propose that this domain participates in the recognition of targeting factors involved in transport to the rhabdomeres.

## Introduction

G protein-coupled receptors (GPCRs) represent the largest family of integral membrane proteins and are the main targets for drug development. They transmit a large variety of extracellular signals to the cell by activating different G proteins. The light receptor rhodopsin is still the best studied GPCR, serving as a prototype, due to its role in vision, but also – historically - due to its abundance in the photoreceptor cell membrane. The three-dimensional structure of endogenous bovine rhodopsin was determined already 10 years ago and served as the basis for understanding also the activation mechanism of GPCRs [1]. Since then, progress with the structure determination of GPCRs was hampered by the low abundance of most GPCRs in their natural membranes and problems with stabilization. Recently the structure of a recombinant bovine rhodopsin expressed in insect cells was determined [2] as well as two structures from the β1 and β2-adrenergic receptors, again from recombinant material [3], [4]. The latter ones were stabilized by different means including fusion proteins, antibody fragments and stabilizing mutations. We established a heterologous system for the overexpression of G protein-coupled receptors (GPCRs) in the eyes of transgenic flies [5]. This system offers a number of advantages compared to conventional eukaryotic expression systems including its low costs and the high quality and homogeneity of the expressed proteins [5]. Ectopic expression of recombinant GPCRs in transgenic *Drosophila* obtained by classical transposition into the genome of fly embryos was driven by an eye-specific promoter element in the photoreceptor cells. Containing microvillar rhabdomeric membranes endogenously filled with rhodopsin, these cells are ideally suited to yield functional GPCRs, as we have exemplarily demonstrated by expression, purification and reconstitution of a metabotropic glutamate receptor able to bind its ligand. Interestingly, by extending this study to a larger number of membrane proteins, we now found that some proteins e.g. the mammalian glutamate receptor, mGluR5, are not spontaneously targeted to the rhabdomeres, but are distributed in other non-ER membranous compartments (data not shown). These findings prompted us to study the molecular mechanisms driving the targeting of the most abundant rhabdomeric protein, the GPCR-prototype rhodopsin.

Faultless transport of rhodopsin from the photoreceptor cell body to the light sensitive membranous compartment of the eye is indispensable for proper visual function and eye development [6], [7]. In human, rhodopsin mutations accounting for its intracellular mislocalization are the most frequent cause of autosomal dominant Retinitis Pigmentosa (RP) [8]–[10], a degenerative retinal pathology characterized by progressive blindness. The most severe forms of RP are provoked by mutations clustered in the rhodopsin C-terminal QVxPA motif [11], which is conserved among vertebrates and has been shown to comprise a binding surface for transport-associated proteins [12], [13]. Deletion or alteration of this motif induced trafficking impairment in both *in vitro* systems and animal models, the latter one accompanied by photoreceptor cell death, displaying its very important role for transport to the membranous rod outer segment (ROS) [14]–[16].

In *Drosophila*, rhodopsin is not only essential for visual responses, but also plays a major role during photoreceptor cell development, being indispensable for the formation and maintenance of its own compartment, the rhabdomere [7], [17]–[19]. The rhabdomere consists of a highly pleated array of microvilli, formed by the apical membrane, which harbors the proteins of the phototransduction signaling cascade. Vesicular transport of rhodopsin to this light-sensitive organelle is known to take place from the trans-Golgi network (TGN) along the actin filamentous *rhabdomere terminal web* (involving the motorprotein MyoV [20]). However, the precise mechanism of rhodopsin trafficking to the rhabdomere remains elusive, and no distinct targeting signal has so far been identified in its amino acid sequence. In both vertebrates and invertebrates the C-terminus has been shown to act as a binding platform for various factors associated with signal transduction [21], [22]. In example, termination of the light-response involves phosphorylation of a conserved serine-rich region in the C-terminus of rhodopsins and binding of arrestin followed by internalization of the complex. As a specific criterion of Class I GPCRs, the C-terminus of rhodopsin comprises a membrane anchored region containing an amphipathic helix (helix 8) and one or more palmitoylated cystein residues, followed by a flexible polar region. In vertebrate rhodopsins this polar region contains the thoroughly investigated QVxPA motif [12]–[14], [23] required for effective targeting to the ROS. Given the importance of this motif, it is surprising to note that it is not conserved among invertebrates.

Here we show that major differences exist concerning the requirements to properly target vertebrate or invertebrate rhodopsins. The extreme C-terminus of *Drosophila* rhodopsin is dispensable, but the adjacent amphipathic region, helix 8, is crucial for correct localization of the receptor and intact eye morphology. In invertebrate rhabdomeric rhodopsins this region contains conserved amino acid residues, which are absent in vertebrate homologs. Helix 8 might therefore act as a binding domain for targeting factors involved in transport to the rhabdomeres.

## Results and Discussion

**Figure 1:**
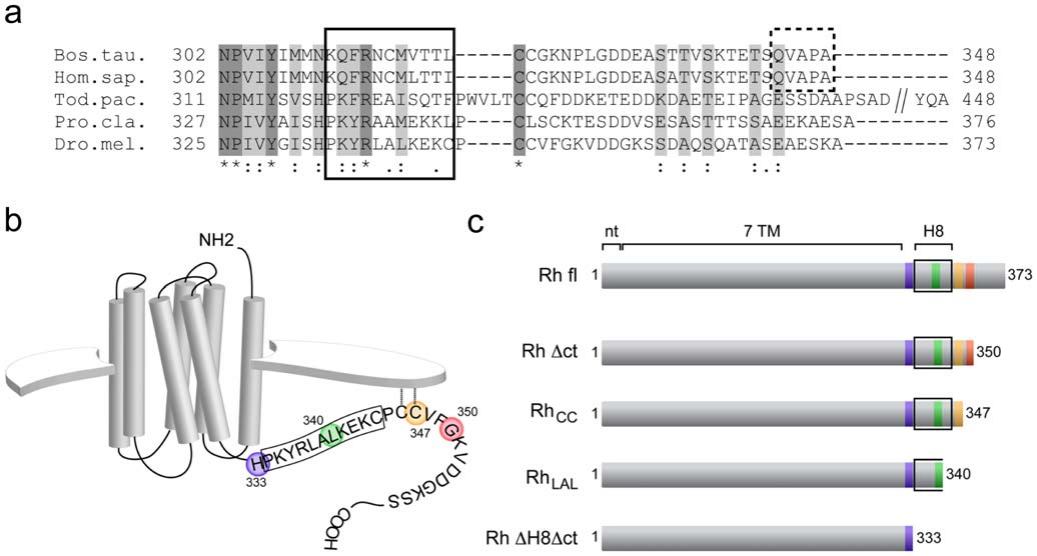
Analysis of the rhodopsin C-terminal region. (a) Sequence alignment of the C-terminus from different species shows that the QVxPA motif of vertebrate rhodopsin is not conserved in invertebrate homologs. Bos.tau: *Bos Taurus*; Hom.sap: *Homo sapiens*; Tod.pac: *Todarodes pacificus (squid)*; Pro.cla: *Procambarus clarkii (crayfish)*; Dro.mel: *Drosophila melanogaster*. Alignment was done with ClustalW2 (EMBL-EBI). Numbers designate amino acid positions. “*” indicates identical (dark grey), “:” conserved (light grey) and “.” semi-conserved amino acid residues with respect to all investigated species. Helix 8 deduced from the structures of bovine and squid rhodopsin [28], [29], [36] is denoted by a continuous black rectangle, QVxPA motif by a dashed black rectangle. “//” indicates position of residues discarded in the representation. (b+c) Schematic representation of *Drosophila* rhodopsin Rh1 variants used in this study. Numbers indicate amino acid positions. Putative helix 8 is denoted by a black box and the last amino acid of respective truncation mutants by colored circles (b) or rectangles (c) (blue: *RhΔctΔH8*, orange: Rh_cc_, green: *Rh_LAL_*, red: *RhΔct*). Putative palmitoylation sites are illustrated by dashed lines linking the cystein residues to the membrane. Abbr. fl: full-length; nt: N-terminus; H8: helix 8.

The comparison of the C-terminal sequences of vertebrate and invertebrate rhodopsin shows that the QVxPA motif is not conserved in invertebrates, which show a significant variability in length and amino acid composition of their extreme C-termini (Fig. 1a). To determine the role of the C-terminal region in the targeting of the fly rhodopsin, we expressed several GFP-fused C-terminally truncated *Drosophila* rhodopsin Rh1 mutants (Fig. 1b–c) in the photoreceptor cells of transgenic flies and studied their ability to reach the rhabdomeres.

**Figure 2:**
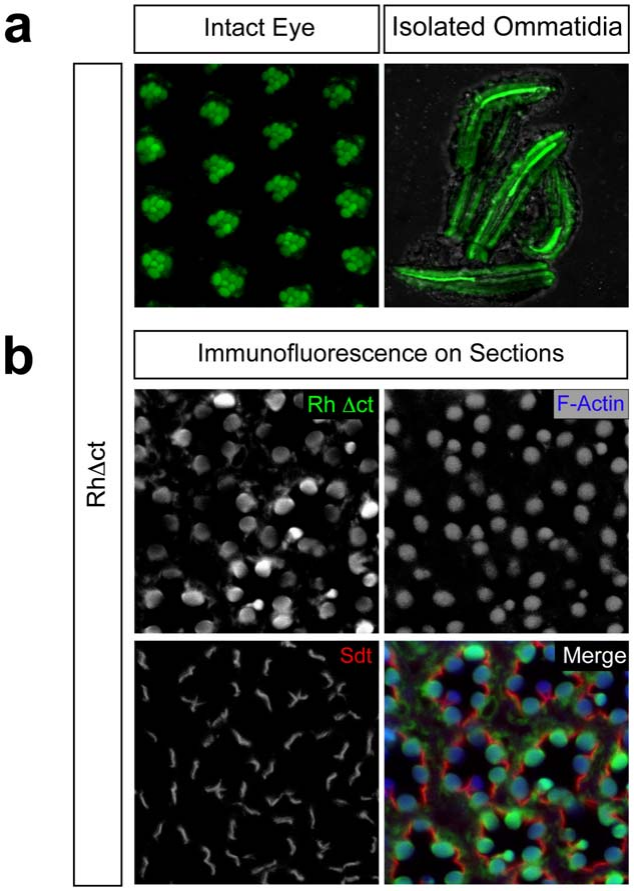
The C-terminally truncated *Drosophila* rhodopsin *RhΔct* localizes to the rhabdomeres. (a) Direct *in vivo* confocal fluorescence microscopy on the intact eye (left) and isolated ommatidia (right) of adult flies expressing GFP-fused *RhΔct* reveals a rhabdomeric localization. (b) Optical cross sections of eyes stained with antibodies against GFP and Stardust (Sdt) show localization of the transgene-encoded protein (green) and stalk membrane (red) respectively. Phalloidin highlights F-Actin-rich rhabdomeres (blue). Merged picture displays extensive colocalization (in turquoise) of the rhodopsin truncation-mutant (green) with F-Actin (blue).

We first removed the last 23 polar amino acids of Rh1 (*RhΔct*) and generated a transgenic fly expressing a truncated rhodopsin variant still containing the amphipathic helix 8 and a putative palmitoylation site. The flies were analyzed using three complementary techniques. Direct *in vivo* analysis of intact eyes and isolated ommatidia revealed strong fluorescence of rhabdomeres (Fig. 2a), as found in flies expressing GFP-fused wildtype rhodopsin [24], [25]. Colocalization of *RhΔct* with F-Actin seen by immunofluorescence on ommatidia cross sections, confirmed its rhabdomeric localization (Fig. 2b). This leads to the conclusion that in contrast to its vertebrate counterparts, *Drosophila* rhodopsin does not require its flexible C-terminus for targeting to the light-sensitive organelle. Interestingly, in a different experimental context, other groups also showed a similar localization of the slightly longer *Rh1*Δ356 construct, which was left uncommented, but supports these findings [26], [27].

**Figure 3:**
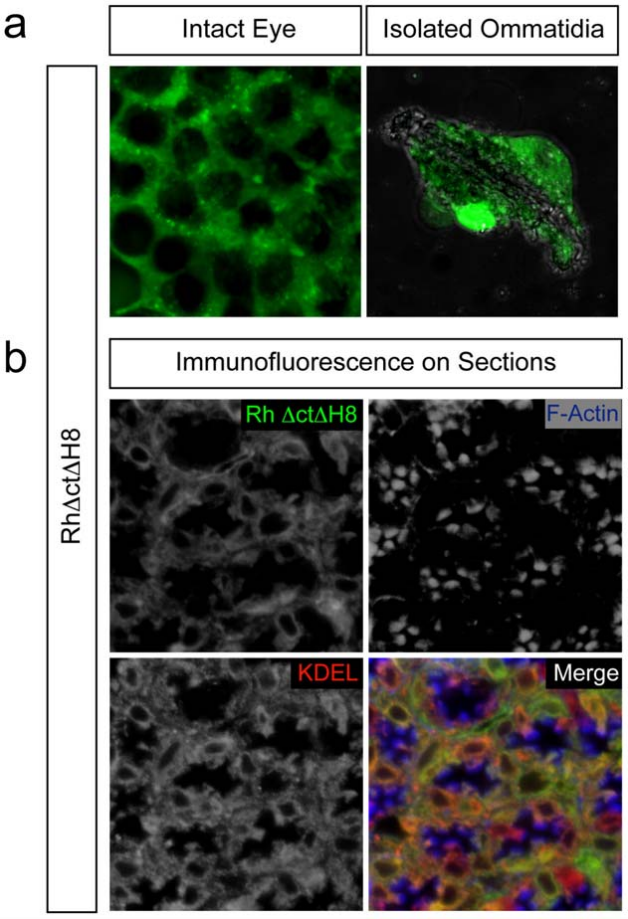
Helix 8 is required for rhabdomere targeting of rhodopsin. (a) Direct *in vivo* confocal fluorescence microscopy on intact eye (left) and isolated ommatidia (right) of adult flies expressing GFP-fused *RhΔctΔH8*, lacking the helix 8 region, shows an extrarhabdomeric distribution-pattern of the truncation-mutant. (b) Optical cross sections, stained with antibodies against GFP and KDEL to show localization of the transgene (green) and ER (red), respectively, reveal its colocalization (orange) with the KDEL antibody, indicating ER-based distribution of the protein. Phalloidin highlights F-Actin-rich rhabdomeres (blue).

**Figure 4:**
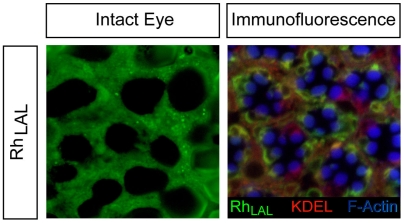
The distal part of Helix 8 is crucial for rhabdomere targeting of rhodopsin. Confocal fluorescence microscopy on intact eye (left) and optical cross sections of an eye expressing *Rh_LAL_* (right), lacking the distal part of helix 8, shows extrarhabdomeric distribution of the rhodopsin variant. The GFP-tagged protein accumulates at the rhabdomere base (in green) and partially colocalizes (in orange) with KDEL, indicating ER-based distribution of the protein (right panel). Optical cross sections were stained with antibodies against GFP and KDEL to show localization of the transgene (green) and ER (red) respectively. Phalloidin highlights F-Actin-rich rhabdomeres (blue).

Additional removal of helix 8 (*RhΔctΔH8* mutant) led to impaired transport of the receptor and a profound alteration of the rhabdomeric structure (Fig. 3). Analysis of both intact eyes and isolated ommatidia showed cytoplasmic accumulation of the protein (Fig. 3a), which was specified endoplasmic reticulum (ER)-based by its colocalization with KDEL in immunofluorescence (Fig. 3b). As ER-retention might be due to misfolding of the receptor [10], we also analyzed a slightly longer Rh1 variant, *Rh_LAL_*, corresponding to a truncation mutant of bovine rhodopsin able to bind retinal and couple to transducin, indicating its proper folding [28], [29]. Preserving the proximal part of helix 8 (Fig. 4), in these flies the rhabdomeric structure was present but *Rh_LAL_* did not colocalize with the rhabdomere marker F-Actin as seen in optical cross sections (Fig. 4b). The shortest Rh1 mutant properly targeted to rhabdomeres (*Rh_CC_*) comprises the entire helix 8 (Fig. S1), showing that the distal part of helix 8 is crucial. It is noteworthy that flies expressing the *Rh_CC_* or *Rh_LAL_* variants develop smaller eyes compared to the *RhΔct* flies, which showed a normal morphology (Fig. S1) as well as a perfect fluorescent pseudopupil (data not shown). As the *Rh_CC_* variant localized in rhabdomeres, it implies that the eye phenotype was not due to a dramatic alteration in rhodopsin trafficking. The palmitoylation site downstream of helix 8 has been shown to be dispensable for the trafficking of both *Drosophila* and vertebrate rhodopsins [30], [31] and therefore the proper targeting of *Rh_CC_* is most likely due to the essential role of helix 8 in the targeting of Rh1.

Analysis of invertebrate opsin sequences showed that rhabdomeric opsins like Rh1 (Fig. S2A) and to a lower extent ciliary opsins, that are homologous to the family of vertebrate visual opsins (Fig. S2B) show high conservation in the proximal part of helix 8 (see the PKY/FR motif). They contain a conserved proline marking the end of helix 8. In the structure of the squid rhodopsin [32], [33], the two conserved prolines induce kinks delimiting helix 8 and their conservation in rhabdomeric opsins suggests common structural features. It is interesting to see the high similarity in the structures of squid rhabdomeric and bovine ciliary rhodopsins despite their low sequence homology [33]. The difference in sequence cannot be attributed to divergence of vertebrate and invertebrate rhodopsins because earlier animal phyla like cnidarians possess both ciliary and rhabdomeric opsins [34]. Moreover the invertebrates honeybee and annelid *Platynereis* have visual rhabdomeric opsins but also non-visual light-detecting opsins that are orthologous to vertebrate ciliary visual opsins [35]. It seems curious that recombinant bovine rhodopsin is properly targeted to the rhabdomeres of a transgenic *Drosophila* [19] since it contains the QVxPA motif but not the residues in helix 8 of *Drosophila* Rh1 which are conserved in rhabdomeric opsins. This may simply indicate that the targeting machinery of *Drosophila melanogaster* can also recognize a targeting motif in the bovine ciliary rhodopsin. Conversely, mammals have a rhabdomeric-like opsin in the retina, melanopsin, playing a role in non-visual responses to light [36], [37]. Melanopsin contains the conserved PKY/FR motif as well as the proline terminating helix 8 as described here for *Drosophila*. A closer inspection of rhabdomeric visual opsins from insects shows that helix 8 is highly conserved (see the PKYRxxLxxR/K motif), but not the very C-terminus of the protein (Fig. S2C). While the proximal part of helix 8 is important in signal transduction in vertebrates [38], [39] and also possibly in invertebrates, in invertebrates the distal part of helix 8 might play a role in the trafficking process. Whether this involves structural requirements e.g. helix formation still needs to be shown.

In summary, our data show that helix 8 of *Drosophila* rhodopsin and not the very C-terminus is essential for proper targeting to the rhabdomere. In contrast, vertebrate rhodopsin shows mislocalization already in the absence of the C-terminal QVxPA motif [14], [40]–[42]. Mazelova et al. recently identified an Arf4 GTPase based targeting complex specifically recognizing the VxPx consensus and thus allowing transport of vertebrate rhodopsin to the ciliary ROS [43]. The consensus sequence is also found in other ciliary membrane proteins, but is absent in rhabdomeric rhodopsin homologs. Together with our findings, this strongly suggests major differences in the targeting of the two rhodopsin families to their respective membranes. There may be a different mechanism responsible for selection and packaging of the rhabdomeric homologs. If during targeting they also interact with a GTPase, they might use helix 8 instead of the C-terminus. Based on our data it is tempting to speculate that this interaction involves conserved residues present at the distal part of helix 8. Ciliary vertebrate and rhabdomeric invertebrate visual rhodopsins are known to activate different G proteins, involve distinct signaling cascades and possess an individual structural fingerprint [1], [32], [33], [revised in 44]. We have shown that they also differ in their targeting sequences. In the future further thorough investigations are needed to understand the exact underlying mechanisms in invertebrates.

## Methods

### Molecular Biology

The *Drosophila* rhodopsin coding sequence was amplified from a *w^1118^ Drosophila* cDNA library (kindly provided by F. Weber, BZH) by PCR using specific primers, and ligated in the pUAST vector (a kind gift from JC Desplan, New York) between EcoRI and NotI restriction sites. Rhodopsin truncation mutants were generated by PCR from this template and cloned alike in pUAST. They encode the following rhodopsin amino acids: *RhΔct*: 1–350, *Rh_cc_* 1–347, *Rh_LAL_* 1–340, *RhΔctΔH8*: 1–333 (see Fig. 1). All of the rhodopsin constructs contain a linker sequence encoding three glycine residues upstream of the NotI restriction site itself coding for three alanines. The nucleotide sequence of eGFP flanked by NotI and XbaI restriction sites was inserted downstream of the rhodopsin and linker sequences. All nucleotide sequences were verified by DNA sequencing.

### Construction and Keeping of Transgenic Flies

Constructs described above were transformed into *Drosophila w^1118^* embryos by the *Vanedis Drosophila injection service* (Norway)[45] by classical P-element transposition. Target-gene expression was driven specifically in the eyes by the Glass Multimer Reporter (GMR)-Gal4 (GMR-Gal4 flies were a kind gift from G. Merdes, ZMBH, Heidelberg) and verified by Western blot analysis using an anti-GFP antibody (Biovision), diluted 1∶2000. All flies were kept at 25° or room temperature on a 12 h day/12 h night cycle.

### Confocal Fluorescence Microscopy

All images were acquired using a Zeiss LSM 510 confocal microscope mounted on an Axiovert 200 inverted microscope equipped with an argon laser beam. Image processing was performed with Adobe Photoshop CS3 according to the guidelines for proper digital image handling [46].

### *In vivo* Microscopy of intact Eyes

Flies were fixed to object slides through a transthoracic needle oriented so that the eyes are placed facing upward. Pictures were taken with a 40× objective lens immersed in oil covering the cornea of the fly. A minimum of eight flies of each genotype were analyzed.

### Isolation of ommatidia and direct *in vivo* Microscopy

At least 10 fly heads of each genotype were bisected, transferred into Schneider cell medium (Gibco) and prepared under a binocular. Using fine needles, retinas were freed from surrounding tissue and fragmented until appropriate in size. Samples were protected by cover slips and instantly analyzed using a 63× objective (NA 0.55).

### Indirect Immunohistochemistry

To verify the *in vivo* data and determine the precise intracellular distribution of the rhodopsin variants, we checked for colocalization with organelle-specific antibodies in immunofluorescence microscopy. Optical cross sections and immunohistochemistry were performed as previously described [47]. The following primary antibodies were used: mouse anti-KDEL (Stressgen) 1∶500 marking the ER, mouse anti-Stardust (Sdt) 1∶400 labeling the stalk membrane that is a part of apical membrane adjacent (basally) to the rhabdomere [48] and rabbit anti-GFP (Invitrogen) 1∶500. Detection was performed by the following corresponding secondary fluorescent antibodies: Cy3 conjugated anti-mouse (Dianova) 1∶200 and Cy2 conjugated anti-rabbit (Dianova) 1∶200. F-Actin of rhabdomeres was stained by Alexa Fluor 660 phalloidin (Molecular Probes) 1∶40.

## Supporting Information

Figure S1 Eye phenotype of the trangenic fly RhCC is not correlated with a defect in rhodopsin trafficking. Eye morphology of one-day old transgenic flies expressing RhΔct (Rh1, 1–350, panel d), RhCC (Rh1, 1–347, panel h) or RhLAL (Rh1, 1–340, panel l)) was analysed in parallel with the localization of the truncated Rh1 transgene-encoded protein in isolated ommatidia. Typical bright field- and fluorescent images of isolated ommatidia obtained by confocal microscopy (objective 60x) are shown in panels [c, g, k] and [a–b, e–f, i–j], respectively. Although both RhΔct and RhCC proteins were localized in rhabdomeres (see panel a–b and e–f, respectively), the fly expressing RhΔct had a normal eye morphology (panel d) while the RhCC fly displays a smaller eye (panel h).
(5.36 MB TIF)

Figure S2 The conserved amino acid residues flanking helix 8 in rhabdomeric opsins are not conserved in ciliary opsins. (a) Sequence alignment of C-terminal regions of rhabdomeric opsins from various phyla of invertebrates using ClustalW2 (EBI, EMBL): Drosophila melanogaster (Dro.mel., Arthropoda), Apis mellifera (Api.mel., Arthropoda), Daphnia pulex (Dap.pul., Crustacean), Schistosoma mansoni (Schi.man., Platyhelminthe), Todarodes pacificus (Tod.pac., Mollusca), Platynereis dumerilii (Plat.dum., Annelida), Lottia gigantea (Lot.gig., Mollusca). Partial sequences are presented from the ultraconserved NPxxY GPCR-consensus motif to the end, except if interrupted (//). Identical (*), conserved (:) and semi-conserved (.) residues are labeled at the bottom of the alignment. The sequence corresponding to helix 8 in squid rhodopsin (Tod.pac.) is underlined. (b) Sequence alignment of C-terminal regions of ciliary opsins from invertebrates: some invertebrates, like the bee and some annelids, have both ciliary and rhabdomeric opsins. Here, the ciliary opsins of Apis mellifera (Bee) (Api.mel., Arthropoda) and Platynereis dumerilii (Plat.dum., Annelida) are aligned in comparison with the earliest animal phylum possessing complex eyes Cladonema radiatum (Cla.rad., Cnidaria). The Drosophila melanogaster (Dro.mel., Arthropoda) sequence is shown in light grey for comparison (the conserved proline is underlined). (c) Sequence alignment of C-terminal regions of 23 rhabdomeric opsins from different insects: Drosophila melanogaster (Dro.me1, Dro.me2 to Dro.me6 are the Rh1 (ninaE), Rh2 to Rh6 opsins, respectively), Calliphora erythrocephala (Cal.ery), Papilio xuthus (Pap.xu1 and Pap.xu2 are Rh2 and Rh5, respectively), Pieris rapae (Pie.rap), Manduca sexta (Man.se1 and Man.se2 are the products of Manop1 and Manop2 genes, respectively), Vanessa cardui (Van.car), Apis mellifera (Api.me1 and Api.me2 are LWSRh1 and LWSRh2 (long wavelength sensitive opsins), respectively and Api.me3 is an UV-sensitive opsin), Cataglyphis bombycinus (Cat.bo1 and Cat.bo2 are a Rh1 and a short wavelength-sensitive opsin, respectively), Tribolium castaneum (Tri.cas), Schistocerca gregaria (Sch.gre), Pediculus humanus (Ped.hum), Camponotus abdominalis (Cam.abd) and Rhodnius prolixus (Rho.pro). Identical (*), conserved (:) and semi-conserved (.) residues are labeled at the bottom of the alignment. The sequence corresponding to helix 8 of Drosophila Rh1, deduced from the squid rhodopsin structure, is underlined.
(0.66 MB TIF)
