# Supplementary figures and images for: Targeting of Drosophila Rhodopsin Requires Helix 8 but Not the Distal C-Terminus
Source: PLoS One. 2009 Jul 2;4(7):e6101. doi: 10.1371/journal.pone.0006101 (PMC2700256; doi:10.1371/journal.pone.0006101)

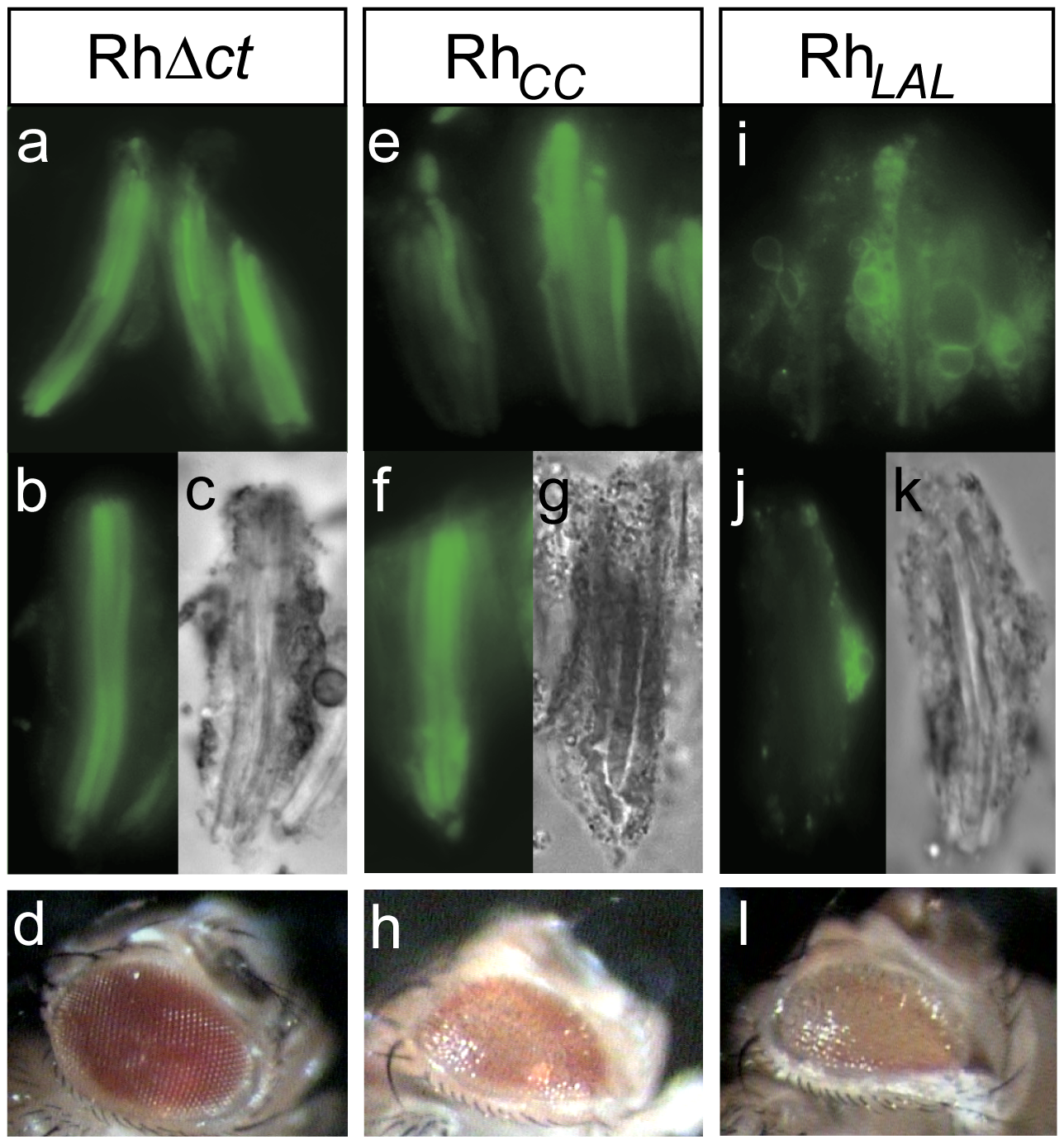

Supplement: Figure S1 — Eye phenotype of the trangenic fly RhCC is not correlated with a defect in rhodopsin trafficking. Eye morphology of one-day old transgenic flies expressing RhΔct (Rh1, 1–350, panel d), RhCC (Rh1, 1–347, panel h) or RhLAL (Rh1, 1–340, panel l)) was analysed in parallel with the localization of the truncated Rh1 transgene-encoded protein in isolated ommatidia. Typical bright field- and fluorescent images of isolated ommatidia obtained by confocal microscopy (objective 60x) are shown in panels [c, g, k] and [a–b, e–f, i–j], respectively. Although both RhΔct and RhCC proteins were localized in rhabdomeres (see panel a–b and e–f, respectively), the fly expressing RhΔct had a normal eye morphology (panel d) while the RhCC fly displays a smaller eye (panel h). (5.36 MB TIF) [file pone.0006101.s001.tif]

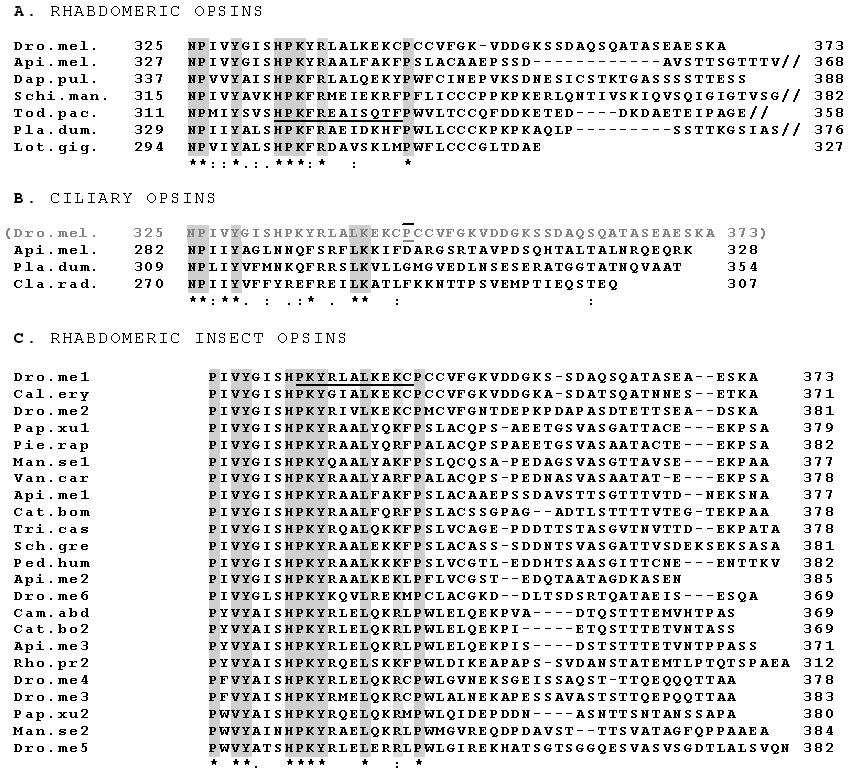

Supplement: Figure S2 — The conserved amino acid residues flanking helix 8 in rhabdomeric opsins are not conserved in ciliary opsins. (a) Sequence alignment of C-terminal regions of rhabdomeric opsins from various phyla of invertebrates using ClustalW2 (EBI, EMBL): Drosophila melanogaster (Dro.mel., Arthropoda), Apis mellifera (Api.mel., Arthropoda), Daphnia pulex (Dap.pul., Crustacean), Schistosoma mansoni (Schi.man., Platyhelminthe), Todarodes pacificus (Tod.pac., Mollusca), Platynereis dumerilii (Plat.dum., Annelida), Lottia gigantea (Lot.gig., Mollusca). Partial sequences are presented from the ultraconserved NPxxY GPCR-consensus motif to the end, except if interrupted (//). Identical (*), conserved (:) and semi-conserved (.) residues are labeled at the bottom of the alignment. The sequence corresponding to helix 8 in squid rhodopsin (Tod.pac.) is underlined. (b) Sequence alignment of C-terminal regions of ciliary opsins from invertebrates: some invertebrates, like the bee and some annelids, have both ciliary and rhabdomeric opsins. Here, the ciliary opsins of Apis mellifera (Bee) (Api.mel., Arthropoda) and Platynereis dumerilii (Plat.dum., Annelida) are aligned in comparison with the earliest animal phylum possessing complex eyes Cladonema radiatum (Cla.rad., Cnidaria). The Drosophila melanogaster (Dro.mel., Arthropoda) sequence is shown in light grey for comparison (the conserved proline is underlined). (c) Sequence alignment of C-terminal regions of 23 rhabdomeric opsins from different insects: Drosophila melanogaster (Dro.me1, Dro.me2 to Dro.me6 are the Rh1 (ninaE), Rh2 to Rh6 opsins, respectively), Calliphora erythrocephala (Cal.ery), Papilio xuthus (Pap.xu1 and Pap.xu2 are Rh2 and Rh5, respectively), Pieris rapae (Pie.rap), Manduca sexta (Man.se1 and Man.se2 are the products of Manop1 and Manop2 genes, respectively), Vanessa cardui (Van.car), Apis mellifera (Api.me1 and Api.me2 are LWSRh1 and LWSRh2 (long wavelength sensitive opsins), respectively and Api.me3 is an UV-sensitive o [file pone.0006101.s002.tif]
